# Real-world effectiveness and prognostic factors of platinum rechallenge in advanced urothelial cancer

**DOI:** 10.1186/s12885-025-15487-w

**Published:** 2025-12-26

**Authors:** Eo Jin Kim, Jwa Hoon Kim, Jinah Chu, Yun-Gyoo Lee, Hyeon-Su Im

**Affiliations:** 1 Division of Hematology/Oncology, Department of Internal Medicine, Kangbuk Samsung Hospital, Sungkyunkwan University School of Medicine, Seoul, Korea; 2 Division of Oncology, Department of Internal Medicine, Korea University Anam Hospital, Korea University College of Medicine, Seoul, Korea; 3 Department of Pathology, Kangbuk Samsung Hospital, Sungkyunkwan University School of Medicine, Seoul, Korea; 4 Division of Hematology and Oncology, Department of Internal Medicine, Ulsan University Hospital, Ulsan University College of Medicine, 25, Daehakbyeongwon-Ro, Dong-Gu, Ulsan, 44033 Republic of Korea

**Keywords:** Urothelial carcinoma, Platinum rechallenge, DNA damage response, Prognostic factors, Real-world data

## Abstract

**Background:**

This study evaluated the real-world effectiveness and prognostic factors of platinum rechallenge therapy in patients with locally advanced or metastatic urothelial carcinoma (la/mUC), with particular attention to DNA damage response (DDR) pathway gene alterations.

**Methods:**

We retrospectively analyzed 78 patients with la/mUC who received platinum rechallenge therapy across three tertiary referral hospitals in Korea. Clinical outcomes, including objective response rate (ORR), progression-free survival (PFS), and overall survival (OS), were assessed. Prognostic factors were identified using univariate and multivariate analyses. The impact of DDR pathway gene alterations on treatment response was also evaluated in patients with available data.

**Results:**

The ORR of platinum rechallenge was 42.3%, with median PFS and OS of 4.7 and 9.0 months, respectively. ORR was numerically higher in ECOG 0–1 versus ≥ 2 (48.2% vs. 27.3%), and in patients without versus with liver metastasis (45.2% vs. 31.2%). Multivariable Cox regression analysis identified ECOG performance status ≥ 2 and liver metastasis as independent adverse prognostic factors. DDR pathway alterations were present in approximately 30% of patients but showed no correlation with ORR, PFS, or OS.

**Conclusion:**

Platinum rechallenge demonstrated meaningful clinical activity in selected patients with la/mUC. Clinical factors such as performance status and liver metastasis may help identify patients most likely to benefit. Although DDR pathway alterations were common, they were not associated with treatment outcomes.

**Supplementary Information:**

The online version contains supplementary material available at 10.1186/s12885-025-15487-w.

## Introduction

Locally advanced or metastatic urothelial carcinoma (la/mUC) has traditionally been treated with platinum-based chemotherapy as the standard first-line treatment [1]. The introduction of immune checkpoint inhibitors (ICIs) and other novel agents has shifted the treatment paradigm for this disease. Pembrolizumab, anti-PD-1 (programmed death-1) antibody, demonstrated a significant overall survival benefit over chemotherapy in patients who progressed after platinum-based chemotherapy, as shown in the phase III KEYNOTE-045 trial [2]. In addition, the JAVELIN Bladder 100 trial showed that avelumab—anti-PD-L1 monoclonal antibody—maintenance therapy prolonged survival in patients without disease progression following first-line platinum-based chemotherapy [3]. Although ICIs have improved outcomes in selected patients with la/mUC, a substantial proportion eventually experience disease progression after an initial period of disease control, and the long-term prognosis still remains poor [4]. In response to this clinical challenge, novel agents such as erdafitinib—a pan-Fibroblast Growth Factor Receptor (FGFR) inhibitor—and enfortumab vedotin—an antibody–drug conjugate targeting Nectin-4—have demonstrated efficacy in patients who failed both platinum-based chemotherapy and ICIs by THOR and EV-301 trial, respectively [5, 6]. More recently, the combination of enfortumab vedotin and pembrolizumab has shown superior survival compared to platinum-based chemotherapy in previously untreated patients, as demonstrated in the EV-302 trial [7].

However, the high cost of these novel therapies poses financial toxicity, limiting access in real-world practice [8]. Furthermore, the application of FGFR inhibitors is also limited by both the costs associated with testing for FGFR alterations and the low prevalence of actionable alterations. As a result, for many patients who experience disease progression after ICI therapy, cytotoxic agents such as paclitaxel or vinflunine remain the only available treatment options. Unfortunately, these agents offer limited survival benefit and are often associated with considerable toxicity [9, 10].

In this context, platinum rechallenge has emerged as a potential alternative strategy. Several retrospective studies have suggested that re-administration of platinum-based chemotherapy may result in meaningful clinical benefits in selected patients. These findings are further supported by real-world evidence demonstrating acceptable response rates and survival outcomes in specific patient subgroups [11–17]. However, current evidence supporting platinum rechallenge in the ICI era is limited, predominantly derived from retrospective studies involving heterogeneous regimens, with only a few small cohorts evaluating exclusively platinum-based rechallenge. In particular, there is currently no data to support optimal patient selection for this strategy.

Therefore, this study aimed to evaluate the clinical efficacy of platinum rechallenge in patients with la/mUC who had previously received platinum-based chemotherapy, with or without subsequent ICIs. Furthermore, we sought to identify subgroups that derive the most benefit from this strategy and to identify prognostic factors that influence treatment outcomes in patients undergoing platinum rechallenge.

## Methods

### Study design and data collection

This multicenter, retrospective cohort study was conducted at three tertiary hospitals in South Korea—Kangbuk Samsung Hospital, Korea University Anam Hospital, and Ulsan University Hospital—and included 78 patients with histologically confirmed, la/mUC who underwent platinum rechallenge therapy between January 2017 and November 2021.

Eligible patients were aged ≥ 18 years and received platinum rechallenge, defined as re-administration of platinum-based chemotherapy after failure of prior platinum therapy. Failure of prior platinum included radiologic progression on or after first-line platinum-based palliative chemotherapy or recurrence within 6 months after completion of neoadjuvant or adjuvant platinum-based chemotherapy. There was no predefined minimum platinum-free (PFI) interval, and regimen repetition was permitted. As this was a retrospective study, the use, timing, and choice of platinum rechallenge reflected real-world clinical judgment rather than predefined criteria. Rechallenge regimens included multi-agent platinum-based combinations such as methotrexate, vinblastine, doxorubicin, and a platinum agent (MVAC; cisplatin or, in selected cases, carboplatin); gemcitabine plus platinum (GP); cisplatin, methotrexate, and vinblastine (CMV); 5-fluorouracil and cisplatin (FP); and paclitaxel, ifosfamide, and cisplatin (TIP). Patients who had previously received perioperative platinum-based chemotherapy were also enrolled in case of disease progression within 6 months. All clinical data were retrospectively obtained from electronic medical records and were anonymized prior to analysis to ensure patient confidentiality.

### Outcomes

All time-to-event outcomes were measured from the initiation of platinum rechallenge, with follow-up continuing until November 2022 and censoring applied at the last available clinical or imaging assessment in patients without events. The primary endpoint was the objective response rate (ORR), defined as the proportion of patients who achieved a complete response (CR) or partial response (PR), as assessed by radiologic imaging and clinical evaluation, according to RECIST version 1.1 criteria. The disease control rate (DCR) was defined as the proportion of patients who achieved CR, PR, or stable disease (SD). Secondary endpoints included progression-free survival (PFS) and overall survival (OS). PFS was defined as the time from platinum rechallenge initiation to documented disease progression or death from any cause, whichever occurred first. OS was defined as the time from platinum rechallenge to death from any cause. Subgroup analyses were conducted based on previously established prognostic factors known to influence outcomes in advanced urothelial carcinoma, including key clinical, pathological, and laboratory variables. Among these, the PFI—defined as the time from completion of prior platinum-based chemotherapy to initiation of platinum rechallenge—was included as an exploratory variable of interest. 

### Next-generation sequencing (NGS), data processing, and pathway analysis

An exploratory subgroup analysis was conducted in patients with available NGS data to assess the relationship between somatic alterations in DDR pathway genes and clinical outcomes following platinum rechallenge. Tumor samples obtained at the time of initial diagnosis were formalin-fixed, paraffin-embedded (FFPE) and used for targeted next-generation sequencing. Prior to sequencing, extracted DNA underwent quality control, including fragment size confirmation and library quantification. Sequencing was conducted using institution-specific gene panels, and platform-specific quality control (QC) metrics such as mean depth, on-target rate were assessed to ensure data quality. Each institution applied its respective NGS platform: Ulsan University Hospital used the Oncomine Comprehensive Assay v3 [18], Kangbuk Samsung Hospital used the CancerSCAN compact^Ⓡ^ panel [19] and Korea University Anam Hospital utilized the Illumina TruSight Oncology 500 NextSeq panel [20].

Twenty-six DDR pathway genes including *BRCA1, BRCA2, ATM, CHEK2, ERCC2, FANCA, PALB2* were identified based on PubMed searches and the NCBI Gene and Biosystems Databases (S1 Table) [21]. Although the three institutions used different NGS panels, one institution's panel included all 26 DDR pathway genes, whereas the other panels covered a subset. Essential genes such as BRCA1, BRCA2, ATM, MLH1, MSH2, MSH6, PMS2, RAD51C, RAD51D, NBN, RAD50, PALB2, CHEK1, and CHEK2 were included in all panels (S2 Table). Genomic variants identified through NGS were classified as pathogenic, likely pathogenic, benign, likely benign, or variants of uncertain significance (VUS), according to ACMG guidelines, ClinVar, and OncoKB annotations, with additional manual review of relevant literature when necessary. For analysis, pathogenic and likely pathogenic alterations were grouped as clinically relevant, while benign, likely benign, and VUS were considered non-actionable. Although pathogenic variants were occasionally detected in DDR genes not uniformly covered across all panels, all patients classified as DDR-altered also harbored pathogenic variants within the essential DDR genes that were consistently included in every panel. This ensured that inter-panel variability did not affect DDR subgroup assignment.

### Statistical analysis

Demographic variables and baseline characteristics were analyzed using descriptive statistics and frequency analysis. Continuous variables are presented as median and interquartile range, whereas categorical variables presented as frequency and percentage. Survival outcomes were analyzed using Kaplan–Meier methods and log-rank tests. Chi-square tests (with Yates’ correction for 2 × 2 tables) and Fisher's exact tests (when expected counts < 5) were performed for categorical variables. Cox proportional hazards models identified prognostic factors, using backward stepwise selection based on the likelihood ratio (*p*-value cutoff = 0.05). All tests were two-sided; a *p*-value < 0.05 was statistically significant.

Statistical analyses were performed with Python 3.13.2 (pandas 2.2.3, NumPy 2.2.6, SciPy 1.15.2, lifelines 0.30.0); figures were generated using matplotlib 3.10.3 and seaborn 0.13.2.

## Results

### Patients

The final cohort included 78 patients who received platinum rechallenge therapy (Table 1). The median age was 67 years (interquartile range [IQR], 61–74), with 66.7% of patients aged 65 years or older. Most patients were male (76.9%) and had pure urothelial carcinoma histology (76.9%). The primary tumor site was the bladder in 44.9% of patients, the ureter in 23.1%, and the renal pelvis in 15.4%, while 16.7% had synchronous involvement of both upper and lower urinary tracts. ICI therapy was administered to 79.5% of patients, with 52.6% receiving it prior to and 26.9% after platinum rechallenge. The median PFI was 2.33 months (IQR, 0.37–4.89), with 79.5% of patients having a PFI less than 6 months. Platinum rechallenge was administered as second-line therapy in 41 patients (52.6%) and as third-line therapy in 26 patients (33.3%); the remaining 11 patients (14.1%) received it in the fourth-line or later setting. The most frequently used rechallenge regimens were MVAC (60.3%) and GP (35.9%), both of which included either cisplatin or carboplatin as the platinum backbone. Overall, 93.6% of patients received cisplatin-based therapy, while only 6.4% received carboplatin.

**Table 1:** Baseline characteristics

Variable	Platinum rechallenge (*n* = 78)
Age, median (IQR)	67.0 (61.0–74.0)
< 65	26 (33.3%)
≥ 65	52 (66.7%)
Sex	
Male	60 (76.9%)
Female	18 (23.1%)
Primary site	
Bladder	35 (44.9%)
Ureter	18 (23.1%)
Renal pelvis	12 (15.4%)
Multiple^a)^	13 (16.7%)
Histologic variants within urothelial carcinoma	
Pure urothelial	60 (76.9%)
Squamous differentiation	14 (17.9%)
Glandular differentiation	2 (2.6%)
Sarcomatoid variant	1 (1.3%)
Micropapillary variant	1 (1.3%)
ECOG performance status	
0–1	56 (71.8%)
≥ 2	22 (28.2%)
Creatinine clearance, median (IQR)	70 (58.3–81.8)
≥ 60 mL/min	56 (71.8%)
< 60 mL/min	22 (28.2%)
Visceral disease	49 (62.8%)
Liver metastasis	16 (20.5%)
Lung metastasis	37 (47.4%)
Bone metastasis	18 (23.1%)
Regimen of prior platinum therapy	
MVAC^b)^	4 (5.1%)
GP^c)^	72 (92.3%)
Other^d)^	2 (2.6%)
Best response to prior platinum therapy	
CR	8 (10.3%)
PR	38 (48.7%)
SD	17 (21.8%)
PD	12 (15.4%)
N/A	3 (3.8%)
PFI, months, median (IQR)	2.33 (0.37–4.89)
< 6 months	62 (79.5%)
≥ 6 months	16 (20.5%)
Immunotherapy at any timepoint	62 (79.5%)
Immunotherapy before platinum rechallenge	41 (52.6%)
Immunotherapy after platinum rechallenge	21 (26.9%)
Line of platinum rechallenge	
2nd line	41 (52.6%)
3rd line	26 (33.3%)
≥ 4th line	11 (14.1%)
Regimen of platinum rechallenge	
Regimen type	
MVAC^b)^	47 (60.3%)
GP^c)^	28 (35.9%)
Other^d)^	3 (3.8%)
Type of platinum	
Cisplatin-containing regimen	73 (93.6%)
Carboplatin-containing regimen	5 (6.4%)

*IQR* interquartile range, *ECOG* Eastern Cooperative Oncology Group, *CR* complete response, *PR* partial response, *SD* stable disease, *PD* progressive disease, *N/A* not available, *PFI* platinum-free interval

^a^Patients with synchronous tumors involving two or more sites (e.g., bladder and ureter; bladder and renal pelvis)

^b^Methotrexate/vinblastine/doxorubicin (Adriamycin)/cisplatin; includes carboplatin-modified MVAC (Carbo-MVAC)

^c^Gemcitabine/platinum; includes gemcitabine plus cisplatin or carboplatin

^d^FP (fluorouracil/platinum); TIP (paclitaxel/ifosfamide/cisplatin); CMV (cisplatin/methotrexate/vinblastine)

**Table 2:** Effectiveness outcomes with platinum rechallenge

	**Platinum rechallenge (** ***n*** ** = 78)**
Best response	
CR (%)	6 (7.7%)
PR (%)	27 (34.6%)
SD (%)	16 (20.5%)
PD (%)	23 (29.5%)
N/A (%)	6 (7.7%)
Object response rate	33 (42.3%)
Disease control rate	49 (62.8%)
Median PFS, months (95% CI)	4.7 (2.8–6.0)
6-month PFS rate, % (95% CI)	36.5 (24.0–46.1)
12-month PFS rate, % (95% CI)	12.4 (4.5–19.8)
Median OS, months (95% CI)	9.0 (8.1–13.8)
6-month OS rate, % (95% CI)	71.7 (57.9–79.5)
12-month OS rate, % (95% CI)	40.9 (27.4–50.7)

*CR* complete response, *PR* partial response, *SD* stable disease, *PD* progressive disease, *N/A* not available, *PFS* progression-free survival, *CI* confidence interval, *OS* overall survival

**Fig. 1:**
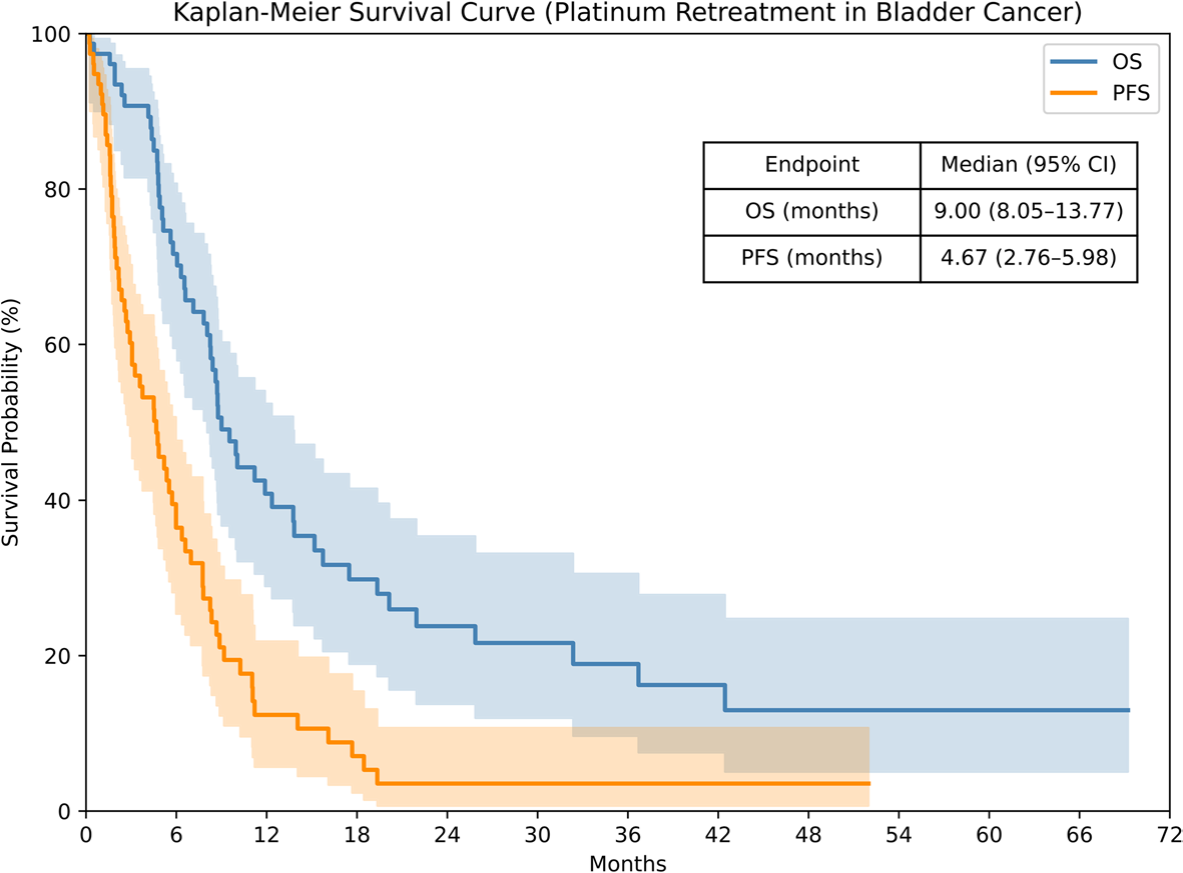
Survival outcomes with platinum rechallenge. OS, overall survival; PFS, progression-free survival

### Subgroup analysis for ORR

**Fig. 2:**
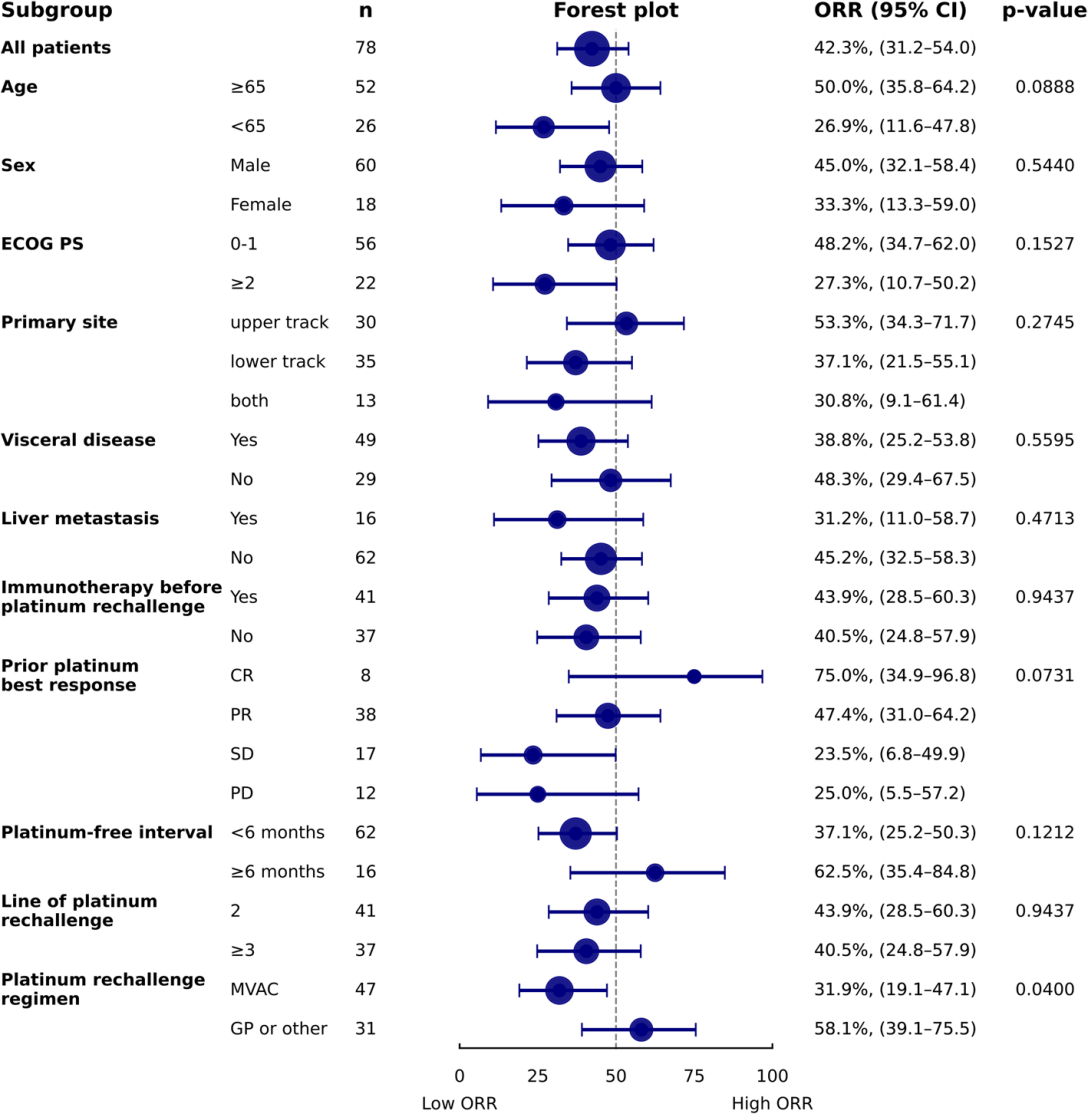
Forest plot of objective response rate in subgroup analyses. ORR, objective response rate; CI, confidence interval; ECOG PS, Eastern Cooperative Oncology Group Performance Status

There were no significant differences in ORR according to each potential subgroup. Despite numerical differences, ORRs were 75%, 47.4%, 23.5%, and 25.0% in patients showing prior CR, PR, SD, and PD with prior platinum chemotherapy. Patients with a PFI ≥ 6 months had a higher ORR than those with a shorter PFI (< 6 months; 62.5% vs. 37.1%). Furthermore, no meaningful differences in ORR were observed according to prior ICI exposure (43.9% vs. 40.5%) or line of rechallenge (43.9% in second-line vs. 40.5% in later lines) (Fig. 2). When analyzing efficacy based on regimen consistency between prior platinum-based chemotherapy and platinum rechallenge, the ORR was significantly higher in the no-regimen-change group compared to the regimen-change group (63.0% vs. 31.4%; *p* = 0.015) (S3 Table).

### Prognostic factors and survival outcomes

**Fig. 3:**
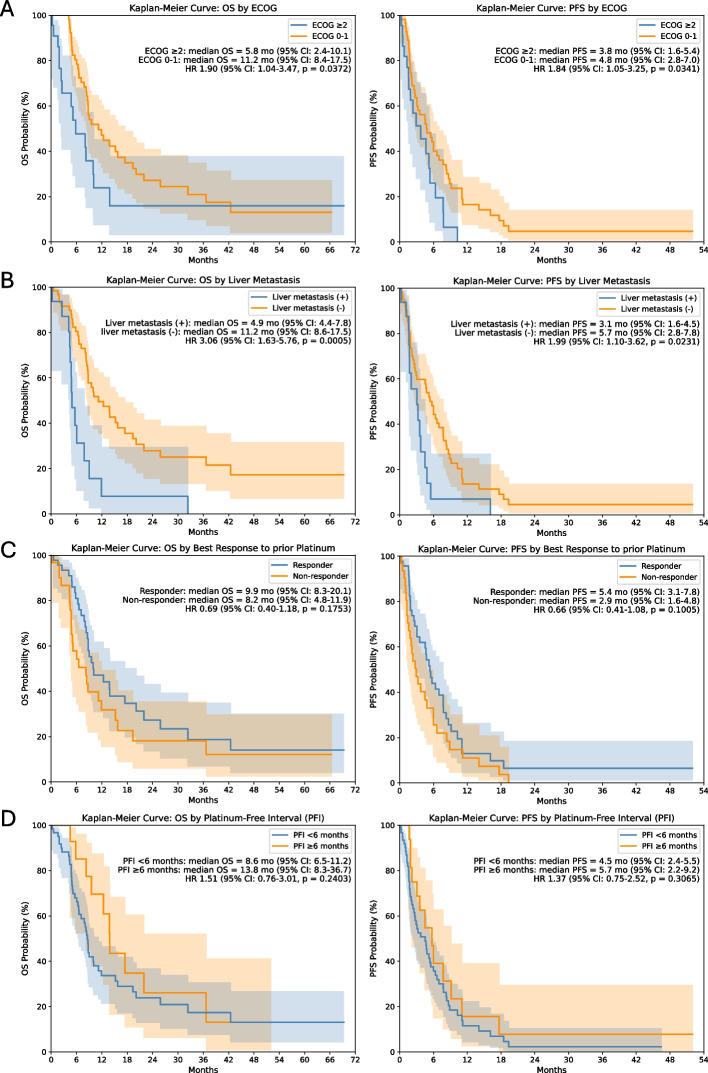
Subgroup survival outcomes with platinum rechallenge. Kaplan–Meier Curve for overall survival and progression-free survival according to (**A**) Eastern Cooperative Oncology Group performance status, (**B**) presence of liver metastasis (**C**) best response to prior platinum-based chemotherapy, (**D**) platinum-free interval. OS, overall survival; PFS, progression-free survival; ECOG, Eastern Cooperative Oncology Group; HR, hazard ratio; CI, confidence interval; PFI, platinum-free interval

Independent prognostic factors for OS and PFS were identified through analyses (Table 3). For OS, significant adverse prognostic factors included ECOG performance status ≥ 2 (HR, 2.26; 95% CI, 1.21–4.22; median OS, 5.8 vs. 11.2 months; *p* = 0.010), liver metastasis (HR, 3.64; 95% CI, 1.88–7.59; median OS, 4.9 vs. 11.2 months; *p* < 0.001), and platinum rechallenge administered at third-line or later (HR, 2.09; 95% CI, 1.20–3.65; median OS, 6.6 vs. 13.8 months; *p* = 0.009). For PFS, analyses consistently identified ECOG performance status ≥ 2 (HR, 2.22; 95% CI, 1.24–3.97; median PFS, 3.8 vs. 4.8 months; *p* = 0.008) and liver metastasis (HR, 2.12; 95% CI, 1.16–3.88; median PFS, 3.1 vs. 5.7 months; *p* = 0.015) as independent predictors of poorer outcomes. Additionally, achieving a response to prior platinum-based chemotherapy was significantly associated with longer PFS (HR, 0.59; 95% CI, 0.36–0.98; median PFS, 5.4 vs. 2.9 months; *p* = 0.040). Other variables, such as the type of platinum rechallenge regimen (MVAC vs. GP or other regimens), regimen consistency, PFI, and prior ICI exposure were significantly associated with neither OS nor PFS (Fig. 3 and S1 Fig.).

**Table 3:** Univariate and multivariate analyses of survival outcomes after platinum rechallenge

**Variable**	Univariable			Multivariable		
	**HR**	**95% CI**	***p*-value**	**HR**	**95% CI**	***p*-value**
**Progression-Free Survival**						
Age ≥ 65 (vs. < 65)	0.88	0.53–1.45	0.618			
Male (vs. female)	1.67	0.91–3.08	0.101			
Pathologic variants (vs. pure urothelial)	1.08	0.60–1.93	0.801			
ECOG ≥ 2 (vs. 0–1)	1.84	1.05–3.25	0.034	2.22	1.24–3.97	0.008
Liver metastasis	1.99	1.10–3.62	0.023	2.12	1.16–3.88	0.015
Prior platinum responder (vs. non-responder)	0.66	0.41–1.08	0.101	0.59	0.36–0.98	0.040
PFI < 6 months (vs. ≥ 6 months)	1.37	0.75–2.52	0.307			
Platinum rechallenge regimen: MVAC^a)^ (vs. GP^b)^ or other^c)^)	1.59	0.95–2.64	0.075			
Immunotherapy before platinum rechallenge	1.54	0.93–2.55	0.094			
Line of platinum rechallenge ≥ 3 (vs. 2)	1.47	0.90–2.39	0.120			
**Overall Survival**						
Age ≥ 65 (vs. < 65)	1.04	0.60–1.82	0.884			
Male (vs. female)	1.46	0.75–2.86	0.270			
Pathologic variants (vs. pure urothelial)	1.27	0.66–2.43	0.465			
ECOG ≥ 2 (vs. 0–1)	1.90	1.04–3.47	0.037	2.26	1.21–4.22	0.010
Liver metastasis	3.06	1.63–5.76	< 0.001	3.64	1.88–7.59	< 0.001
Prior platinum responder (vs. non-responder)	0.69	0.40–1.18	0.175			
PFI < 6 months (vs. ≥ 6 months)	1.51	0.76–3.01	0.240			
Platinum rechallenge regimen: MVAC^a)^ (vs. GP^b)^ or other^c)^)	1.71	0.97–3.04	0.065			
Immunotherapy before platinum rechallenge	1.92	1.10–3.34	0.021			
Line of platinum rechallenge ≥ 3 (vs. 2)	2.12	1.23–3.68	0.007	2.09	1.20–3.65	0.009

*HR* hazard ratio, *CI* confidence interval, *ECOG* Eastern Cooperative Oncology Group, *PFI* platinum-free interval

^a^Methotrexate/vinblastine/doxorubicin (Adriamycin)/cisplatin; includes carboplatin-modified MVAC (Carbo-MVAC)

^b^Gemcitabine/platinum; includes gemcitabine plus cisplatin or carboplatin

^c^FP (fluorouracil/platinum); TIP (paclitaxel/ifosfamide/cisplatin); CMV (cisplatin/methotrexate/vinblastine)

### Clinical outcomes according to the sequence of ICI administration

Subgroup analyses according to the sequence of ICI administration revealed differences in ORR across the three groups: 56.2% in patients without ICI exposure, 43.9% in those who received ICI before platinum rechallenge, and 28.6% in those who received ICI after platinum rechallenge (S4 Table). For OS, ICI administration after platinum rechallenge was associated with longer survival compared with ICI administration before rechallenge (HR 0.46; 95% CI, 0.24–0.88). In contrast, PFS was similar between the ICI-before and ICI-after groups (HR 1.01; 95% CI, 0.58–1.75) (S2 Fig.).

### Complete responders to platinum rechallenge

Among the 78 patients who underwent platinum rechallenge therapy, six (7.7%) achieved a CR. The baseline characteristics and outcomes of these outlier patients are summarized in Table 4. Age and gender distributions were similar to those of the overall patient group. The primary tumor site included bladder (*n* = 2), upper urinary tract (*n* = 3), and multiple sites (*n* = 1). All had pure urothelial histology, ECOG performance status 0–1, and no liver metastasis; four patients had visceral metastasis. Two patients had undergone ICI prior to platinum rechallenge, and two received ICI afterward. Response to prior platinum therapy was CR in four patients (66.7%), PR in one patient (16.7%), and unevaluable in one patient due to no measurable lesion after previous palliative resection. PFI was short (< 6 months; range, 0.2–2.3 months) in three patients and long (≥ 6 months; range, 19.8–43.8 months) in the remaining three. After platinum rechallenge, PFS ranged from 2.6 to 17.7 months (one patient remained progression-free at data cutoff), and OS ranged from 4.5 to 36.7 months, with three patients alive at the data cutoff.

**Table 4:** Characteristics and clinical outcomes of complete responders to platinum rechallenge therapy

Age	Sex	Primary site	Histologic variants	ECOG	Liver metastasis	Immunotherapy before platinum rechallenge	Immunotherapy after platinum rechallenge	Response to prior platinum therapy	Line of platinum rechallenge	PFI (months)	PFS (months)	OS (months)
≥ 65	Female	Multiple	Pure urothelial	1	no	yes	no	CR	3	2.3	2.6	10.8^b)^
≥ 65	Female	Bladder	Pure urothelial	1	no	no	no	CR	2	19.8	4.5^a)^	4.5^b)^
≥ 65	Male	Bladder	Pure urothelial	1	no	no	yes	PR	2	0.2	4.8	8.7
< 65	Male	Upper tract	Pure urothelial	0	no	no	yes	CR	2	21.4	5.7	28.6^b)^
≥ 65	Female	Upper tract	Pure urothelial	1	no	no	no	N/A^c)^	2	43.8	17.7	36.7
< 65	Male	Upper tract	Pure urothelial	0	no	yes	no	CR	3	2.0	11.0	20.1

*ECOG* Eastern Cooperative Oncology Group, *PFI* platinum-free interval, *PFS* progression-free survival, *OS* overall survival, *CR* complete response, *PR* partial response, *N/A* not available

^a^Patient without disease progression at the time of data cutoff

^b^Patients alive at the time of data cutoff

^c^The patient underwent palliative resection before prior platinum chemotherapy and had no evaluable cancer lesion at the time of chemotherapy

### Relationship to DDR pathway gene alterations

**Fig. 4:**
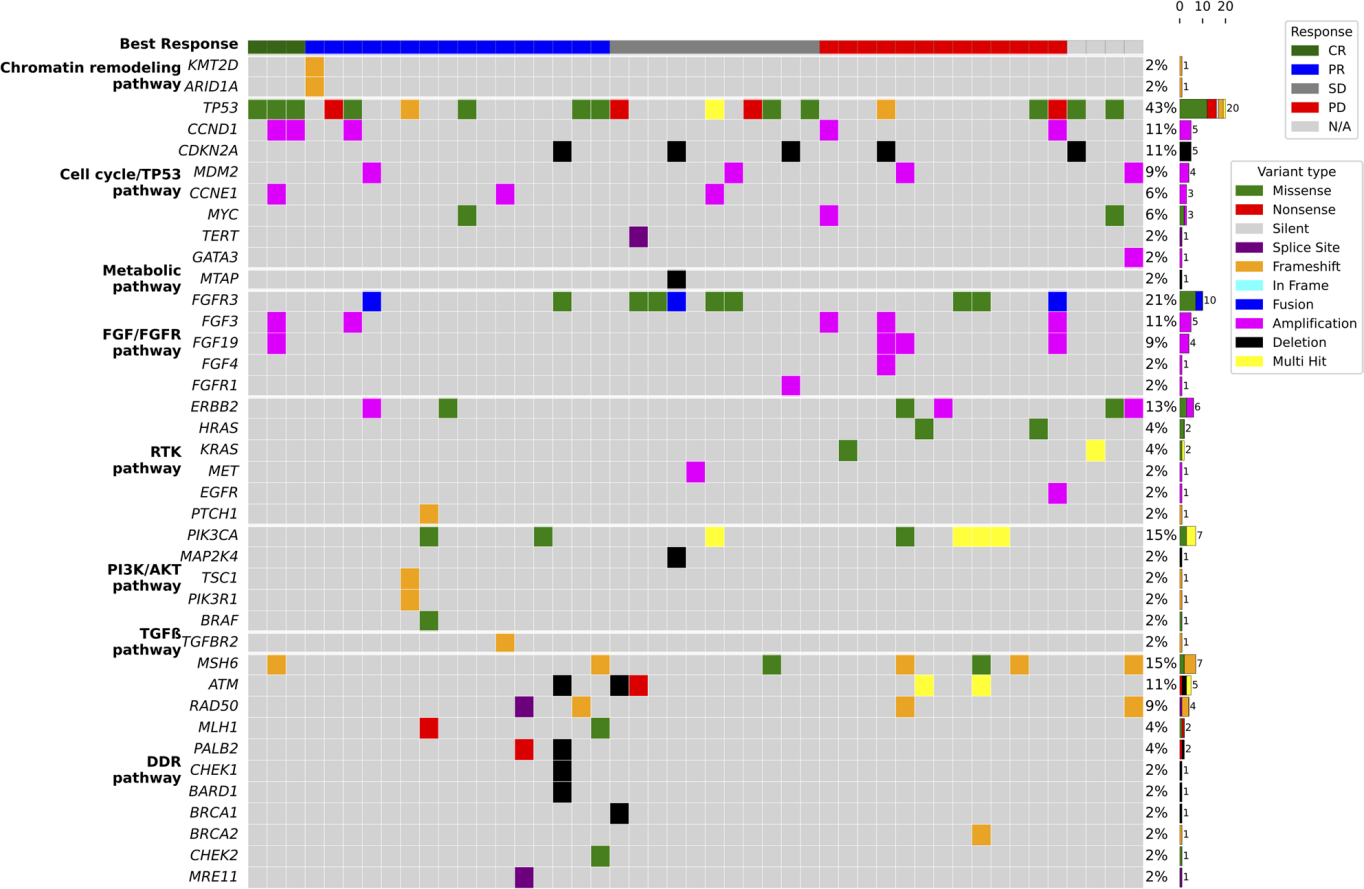
Oncoprint of somatic genetic alterations in patients with available NGS data. CR, complete response; PR, partial response; SD, stable disease; PD, progressive disease; N/A, not available

**Fig. 5:**
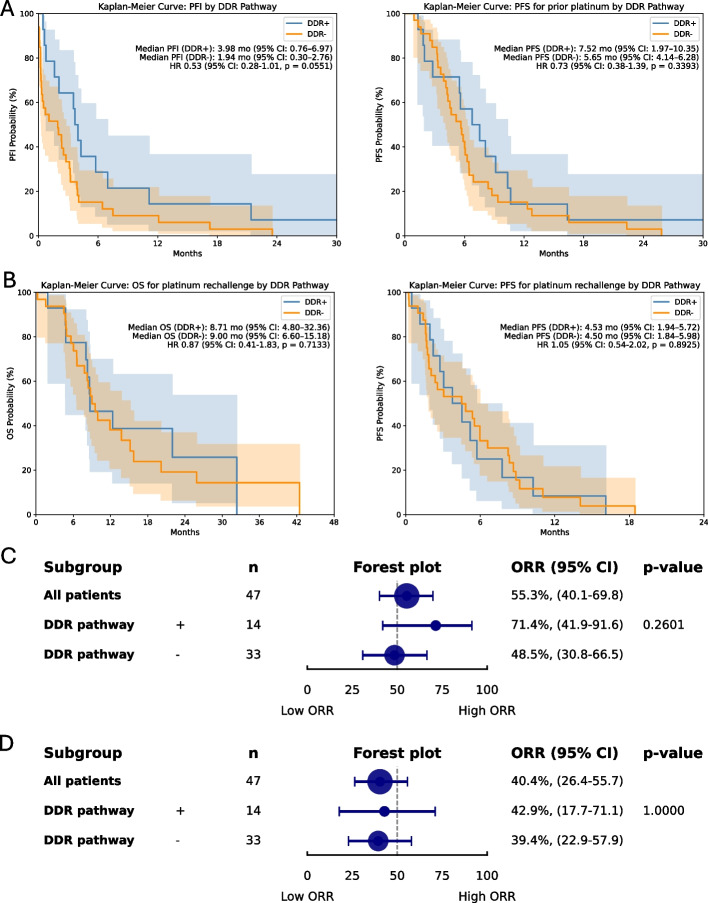
Survival outcomes and objective response rate according to DDR pathway status. (**A**) Kaplan–Meier curves of PFI and PFS after prior platinum therapy according to DDR status, (**B**) Kaplan–Meier curves of OS and PFS after platinum rechallenge according to DDR status, (**C**) Forest plot of ORR to prior platinum therapy according to DDR status, (**D**) Forest plot of ORR to platinum rechallenge according to DDR status. PFI, platinum-free interval; PFS, progression-free survival; OS, overall survival; ORR, objective response rate; CI, confidence interval

Among the 47 patients (60.3% of the cohort) with available NGS data, a total of 125 pathogenic or likely pathogenic variants were identified according to ACMG guidelines. The most frequently altered genes were *TP53* (*n* = 20, 42.6%), *FGFR3* (*n* = 10, 21.3%), *PIK3CA* (*n* = 7, 14.9%), and *MSH6* (*n* = 7, 14.9%). Fourteen patients (29.8%) harbored somatic alterations in DDR pathway genes, most commonly involving *MSH6* (*n* = 7 ), *ATM* (*n* = 5), and *RAD50* (*n* = 4) (Fig. 4). Patients with DDR alterations showed a numerically higher ORR to prior platinum therapy (71.4% vs. 48.5%; *p* = 0.260). Additionally, median PFS (7.5 vs. 5.7 months; HR, 0.73; 95% CI, 0.38–1.39; *p* = 0.339) and median PFI (4.0 vs. 1.9 months; HR, 0.53; 95% CI, 0.28–1.01; *p* = 0.055) for prior platinum-based chemotherapy were numerically longer in the DDR-altered subgroup, though these differences did not reach statistical significance. With respect to platinum rechallenge, the ORR was comparable between DDR-altered and the DDR-wildtype subgroups (42.9% vs. 39.4%, respectively). Kaplan–Meier analyses similarly revealed no statistically significant differences in survival outcomes between DDR-altered and DDR-wildtype subgroups during rechallenge, with identical median PFS in both groups at 4.5 months (HR, 1.05; 95% CI, 0.54–2.02; *p* = 0.893) and nearly equivalent median OS (8.7 vs. 9.0 months; HR, 0.87; 95% CI, 0.41–1.83; *p* = 0.713) (Fig. 5).

### Safety

Treatment-related adverse events during platinum rechallenge were generally manageable (S5 Table). Overall, 93.6% of patients experienced at least one adverse event, and grade 3–4 events occurred in 69.2%. Common toxicities were anemia (69.2%), neutropenia (65.4%), and thrombocytopenia (60.3%). Febrile neutropenia occurred in 3.8% of patients. Non-hematologic toxicities such as elevated liver enzymes, creatinine elevation, mucositis, diarrhea, and vomiting were mostly grade 1–2, and grade 3–4 non-hematologic toxicities were uncommon (< 3%). No unexpected safety signals were identified.

## Discussion and conclusion

In this multicenter retrospective study, we evaluated the clinical effectiveness of platinum rechallenge in patients with la/mUC who previously received platinum-based chemotherapy. Platinum rechallenge yielded an ORR of 42.3% and DCR of 62.8%, with median OS and PFS of 9.0 and 4.7 months, respectively. Poor ECOG performance status (≥ 2) and liver metastasis were independent predictors of worse OS, while prior response to platinum was associated with improved PFS. Later-line platinum rechallenge was also associated with poorer outcomes, but this finding likely reflects clinical context rather than a true prognostic effect. Notably, all complete responders lacked these adverse prognostic factors and exhibited a favorable clinical profile. These efficacy outcomes align with prior retrospective reports (ORR 28.6–66.7%, median OS 7.9–11.2 months, and median PFS 4.1–7.9 months) [14–17], but our larger cohort (*n* = 78) spanning pre- and post-ICI eras enabled more robust subgroup analyses and greater clinical applicability than earlier small-series studies (*n* = 12–25).

Our analysis indicates that platinum rechallenge may provide clinically meaningful activity in la/mUC patients. The observed efficacy outcomes fell within the broad range reported for agents such as enfortumab vedotin and erdafitinib in EV-301 and THOR [5, 6]; however these indirect comparisons should be interpreted cautiously given the substantial differences between prospective, controlled trials and our retrospective, single-arm rechallenge cohort. These findings are reinforced by recent real-world studies. A propensity-matched analysis found no clear survival advantage of enfortumab vedodtin over platinum rechallenge after ICI therapy [15]. Another study reported that platinum rechallenge or clinical trials (primarily involving enfortumab vedotin or erdafitinib) was associated with better outcomes than taxane- or pemetrexed-based chemotherapy in third-line settings [11]. Thus, platinum rechallenge remains a viable and effective alternative whereas access to novel therapies is limited by cost or regulations.

Analysis based on ICI exposure before platinum rechallenge provided insight into its potential impact on treatment outcomes. A prior retrospective study reported no differences in ORR or PFS between chemotherapy rechallenge administered after versus before ICI therapy (ORR: 29.2% vs. 31.0%; median PFS: 4.9 vs. 4.6 months; *p* = 0.62), although the inclusion of non–platinum regimens such as taxanes and vinflunine limits the platinum-specific relevance of those findings [13]. In our cohort, ORR was similar between patients with and without prior ICI exposure, while OS and PFS appeared numerically worse in the ICI-exposed group (S1 Fig.); however, these differences were not statistically significant after adjustment for other clinical covariates in multivariable analysis. This pattern likely reflects differences in clinical context: ICI-unexposed patients tended to receive platinum rechallenge earlier in the disease course, most often as a second-line treatment, with relatively preserved performance status and lower cumulative treatment-related toxicity, whereas ICI-exposed patients more frequently received rechallenge in later lines. To further explore the influence of ICI administration in treatment sequence relative to platinum rechallenge, we performed a three-way comparison of patients with no ICI exposure, ICI administered before rechallenge, and ICI administered after rechallenge (S2 Fig. and S4 Table). PFS after platinum rechallenge was similar between patients who received ICI before and after rechallenge, whereas OS appeared longer in those who received ICI afterward compared with those who received it beforehand. Given the absence of a corresponding PFS advantage, this OS difference is more plausibly attributable to differences in clinical context including disease biology, treatment sequencing, and subsequent ICI therapy rather than to a direct interaction between ICI administration sequence and platinum sensitivity.

In the present study, multivariable analysis identified ECOG performance status and the presence of liver metastases as independent prognostic factors for OS. ECOG performance status (≥ 2), liver metastases, and lack of response to prior platinum-based chemotherapy were associated with inferior PFS. These findings are consistent with previously established prognostic factors for systemic chemotherapy in la/mUC, highlighting their continued relevance in clinical decision-making and treatment planning [22–25]. Interestingly, although univariable analyses suggested improved outcomes with non-MVAC regimens, these associations were not sustained in multivariable analysis. This pattern likely reflects treatment selection bias, as most patients receiving non-MVAC regimens (87.1%) also underwent regimen reuse and tended to have more favorable baseline characteristics, including better prior platinum response, longer PFI, and earlier lines of therapy. Accordingly, these observation should not be interpreted as evidence of intrinsic superiority of any specific rechallenge regimen.

In our study, somatic alterations in DDR pathway genes were detected in approximately 30% of la/mUC patients with NGS testing. Prior reports have consistently demonstrated that DDR deficiency is associated with increased sensitivity to platinum chemotherapy, owing to impaired DNA repair capacity that amplifies the cytotoxic effects of platinum agents [26, 27]. Consistent with these findings, patients in our DDR-altered subgroup showed numerical trends toward higher ORR and longer PFS and PFI with initial platinum therapy, although these differences did not reach statistical significance. In contrast, DDR status was not associated with ORR, OS, or PFS in the time of platinum rechallenge, suggesting that the chemosensitivity conferred by DDR impairment during initial exposure may not be retained upon re-exposure to platinum. Several mechanisms may account for this observation. Acquired resistance can emerge through secondary reversion mutations restoring BRCA function, reconstitution of homologous recombination, or shifts in DNA repair pathway usage such as increased reliance on non-homologous end joining [28–30]. Furthermore, therapeutic pressure may promote subclonal selection of tumor cell populations with preserved DNA repair capacity, contributing to reduced responsiveness to subsequent platinum rechallenge [31]. Our results support the concept that although DDR-altered tumors exhibit enhanced sensitivity to initial platinum therapy, subsequent exposure to platinum may select for resistant clones. However, given the limited sample size and exploratory nature of these analysis, these findings should be interpreted with caution and warrant validation in larger, prospective cohorts.

Despite the strengths of this study, including its multicenter design and integration of detailed clinical and genomic analyses, several limitations should be acknowledged. First, the retrospective nature of the analysis inevitably introduced selection biases related to treatment decisions, sequencing of systemic therapies, and heterogeneity in ICI use. Second, comprehensive genomic profiling was available for only a subset of patients, and local, institution-specific NGS panels were used rather than a standardized, centralized assay. Inter-institutional differences in sequencing platforms and analytic pipelines may have affected variant detection and interpretation and may have reduced power to identify robust molecular predictors. Third, the absence of a contemporaneous control or comparator group restricts our ability to contextualize the observed outcomes relative to other available treatment options. In addition, heterogeneity in platinum-based regimens across institutions may have introduced treatment variability that may affect the generalizability of our findings. Finally, the modest sample size of our single-arm cohort (*n* = 78) limited our ability to detect potentially clinically meaningful differences, particularly in subgroup and biomarker analyses.

In conclusion, our multicenter retrospective study highlights that platinum rechallenge remains an effective and clinically relevant therapeutic option for patients with la/mUC, even in the era of novel agents. These findings underscore the enduring clinical value of platinum rechallenge as a treatment strategy in the evolving therapeutic landscape. Readily accessible clinical parameters—including ECOG performance status, presence of liver metastases, and response to prior platinum-based chemotherapy—emerge as practical prognostic factors that may assist in selecting appropriate candidates for this strategy. Importantly, DDR pathway alterations, despite their known association with sensitivity to initial platinum chemotherapy, do not appear to predict benefit from platinum rechallenge, suggesting that resistance mechanisms may emerge with repeated platinum exposure. Further prospective studies incorporating standardized genomic profiling are warranted to validate these findings and elucidate the biological basis of acquired resistance to platinum rechallenge in la/mUC.

## Supplementary Information

Supplementary Material 1.

## Data Availability

The datasets generated and/or analyzed during the current study are available in the figshare repository, [https://doi.org/10.6084/m9.figshare.30171253] (https://doi.org/10.6084/m9.figshare.30171253). All relevant clinical and genomic data underlying the findings of this study have been de-identified to protect patient confidentiality. Additional supporting tables and figures are included in the supplementary files.
