# Supplementary material for: Real-world effectiveness and prognostic factors of platinum rechallenge in advanced urothelial cancer
Source: BMC Cancer. 2025 Dec 26;26:185. doi: 10.1186/s12885-025-15487-w (PMC12870190; doi:10.1186/s12885-025-15487-w)
Supplement: Supplementary file 1 — Supplementary Material 1. [file 12885_2025_15487_MOESM1_ESM.docx]

**S1 Table. List of genes included in the DNA damage response (DDR) pathway.**

| **Mismatch repair** | **Homologous  recombination** | **Fanconi anemia** | **Checkpoint** | **Other** |
| --- | --- | --- | --- | --- |
| MLH1 | BRCA1 | FANCF | ATR | GEN1 |
| MSH2 | BRCA2 |  | CHEK1 | PARP1 |
| MSH6 | RAD51 |  | CHEK2 |  |
| PMS2 | RAD51C |  | MDC1 |  |
|  | RAD51D |  |  |  |
|  | XRCC2 |  |  |  |
|  | ATM |  |  |  |
|  | NBN |  |  |  |
|  | MRE11A |  |  |  |
|  | RAD50 |  |  |  |
|  | BRIP1 |  |  |  |
|  | PALB2 |  |  |  |
|  | BAP1 |  |  |  |
|  | BARD1 |  |  |  |
|  | FAM175A |  |  |  |

**S2 Table.** **List of DDR Pathway Genes Included in Each Institutional NGS Panel**

| **Institution** | **Kangbuk Samsung Hospital** | **Korea University Anam Hospital** | **Ulsan University Hospital** |
| --- | --- | --- | --- |
| **NGS panel** | **CancerSCAN compact^Ⓡ^** | **Illumina TruSight Oncology 500 NextSeq** | **Oncomine Comprehensive Assay v3** |
| **Mismatch repair** | MLH1 | MLH1 | MLH1 |
|  | MSH2 | MSH2 | MSH2 |
|  | MSH6 | MSH6 | MSH6 |
|  | PMS2 | PMS2 | PMS2 |
| **Homologous  recombination** | BRCA1 | BRCA1 | BRCA1 |
|  | BRCA2 | BRCA2 | BRCA2 |
|  | RAD51C | RAD51 | RAD51 |
|  | RAD51C | RAD51C | RAD51C |
|  | RAD51D | RAD51D | RAD51D |
|  | ATM | ATM | ATM |
|  | NBN | NBN | NBN |
|  | RAD50 | RAD50 | RAD50 |
|  | PALB2 | PALB2 | PALB2 |
|  |  | RAD51 | RAD51 |
|  |  | BAP1 | BAP1 |
|  | BRIP1 | BRIP1 |  |
|  | BARD1 | BARD1 |  |
|  |  | MEE11A |  |
|  |  | XRCC2 |  |
| **Fanconi anemia** | FANCF | FANCF |  |
| **Checkpoint** | CHEK1 | CHEK1 | CHEK1 |
|  | CHEK2 | CHEK2 | CHEK2 |
|  |  | ATR | ATR |
|  |  | MDC1 |  |
| **Other** |  | GEN1 |  |
|  |  | PARP1 |  |

**S3 Table. Effectiveness outcomes by regimen of platinum rechallenge.**

|  |  | **Best response** | | | | | |  |
| --- | --- | --- | --- | --- | --- | --- | --- | --- |
|  | **n** | **CR** | **PR** | **SD** | **PD** | **N/A** | **ORR** | **p-value** |
| **Total** | 78 | 6 (7.7%) | 27 (34.6%) | 16 (20.5%) | 23 (29.5%) | 6 (7.7%) | 33 (42.3%) |  |
| **Regimen change** | 51 | 2 (3.9%) | 14 (27.5%) | 12 (23.5%) | 18 (35.3%) | 5 (9.8%) | 16 (31.4%) | 0.015 |
| **No regimen change** | 27 | 4 (14.8%) | 13 (48.1%) | 4 (14.8%) | 5 (18.5%) | 1 (3.7%) | 17 (63.0%) |  |
| **Regimen sequence details** |  |  |  |  |  |  |  |  |
| MVAC† → MVAC† | 0 | 0 (0.0%) | 0 (0.0%) | 0 (0.0%) | 0 (0.0%) | 0 (0.0%) | 0 (0.0%) | 0.019 |
| MVAC† → GP‡ or other* | 4 | 0 (0.0%) | 1 (25.0%) | 2 (50.0%) | 1 (25.0%) | 0 (0.0%) | 1 (25.0%) |  |
| GP‡ or other* → MVAC† | 47 | 2 (4.3%) | 13 (27.7%) | 10 (21.3%) | 17 (36.2%) | 5 (10.6%) | 15 (31.9%) |  |
| GP‡ or other* →  GP‡ or other* | 27 | 4 (14.8%) | 13 (48.1%) | 4 (14.8%) | 5 (18.5%) | 1 (3.7%) | 17 (63.0%) |  |
| †MVAC: Methotrexate/vinblastine/doxorubicin (Adriamycin)/cisplatin; includes carboplatin-modified MVAC (Carbo-MVAC). ‡GP: Gemcitabine/platinum; includes gemcitabine plus cisplatin or carboplatin. *Other: FP (fluorouracil/platinum), TIP (paclitaxel/ifosfamide/cisplatin), CMV (cisplatin/methotrexate/vinblastine). Abbreviations: CR, complete response; PR, partial response; SD, stable disease; PD, progressive disease; N/A, not available; ORR, overall response rate; DCR, disease control rate. | | | | | | | | |

**S4 Table. Objective response rate (ORR) after platinum rechallenge according to the timing of immunotherapy exposure.**

|  | **N** | **Responders**  **(CR + PR)** | **ORR (95% CI)** |
| --- | --- | --- | --- |
| **No immunotherapy** | 16 | 9 | 56.2% (29.9–80.2) |
| **Immunotherapy before platinum rechallenge** | 41 | 18 | 43.9% (28.5–60.3) |
| **Immunotherapy after platinum rechallenge** | 21 | 6 | 28.6% (11.3–52.2) |

**S5 Table. Treatment-related adverse events during platinum rechallenge.**

| **Adverse event** | **All grade, n (%)** | **Grade 3–4, n (%)** |
| --- | --- | --- |
| **Any adverse event** | 73 (93.6%) | 54 (69.2%) |
| **Neutropenia** | 51 (65.4%) | 37 (47.4%) |
| **Febrile neutropenia** | 3 (3.8%) | 3 (3.8%) |
| **Anemia** | 54 (69.2%) | 27 (34.6%) |
| **Thrombocytopenia** | 47 (60.3%) | 13 (16.7%) |
| **Elevated AST/ALT** | 17 (21.8%) | 2 (2.6%) |
| **Hyperbilirubinemia** | 2 (2.6%) | 1 (1.3%) |
| **Creatinine elevation** | 28 (35.9%) | 2 (2.6%) |
| **Vomiting** | 22 (28.2%) | 0 (0%) |
| **Diarrhea** | 21 (26.9%) | 0 (0%) |
| **Oral mucositis** | 28 (35.9%) | 2 (2.6%) |

**S1 Fig. Kaplan–Meier curves for OS and PFS after platinum rechallenge according to (A) platinum rechallenge regimen (MVAC vs. GP or other regimens), (B) treatment regimen change, (C) line of platinum rechallenge, and (D) prior exposure to immune checkpoint inhibitor therapy.**


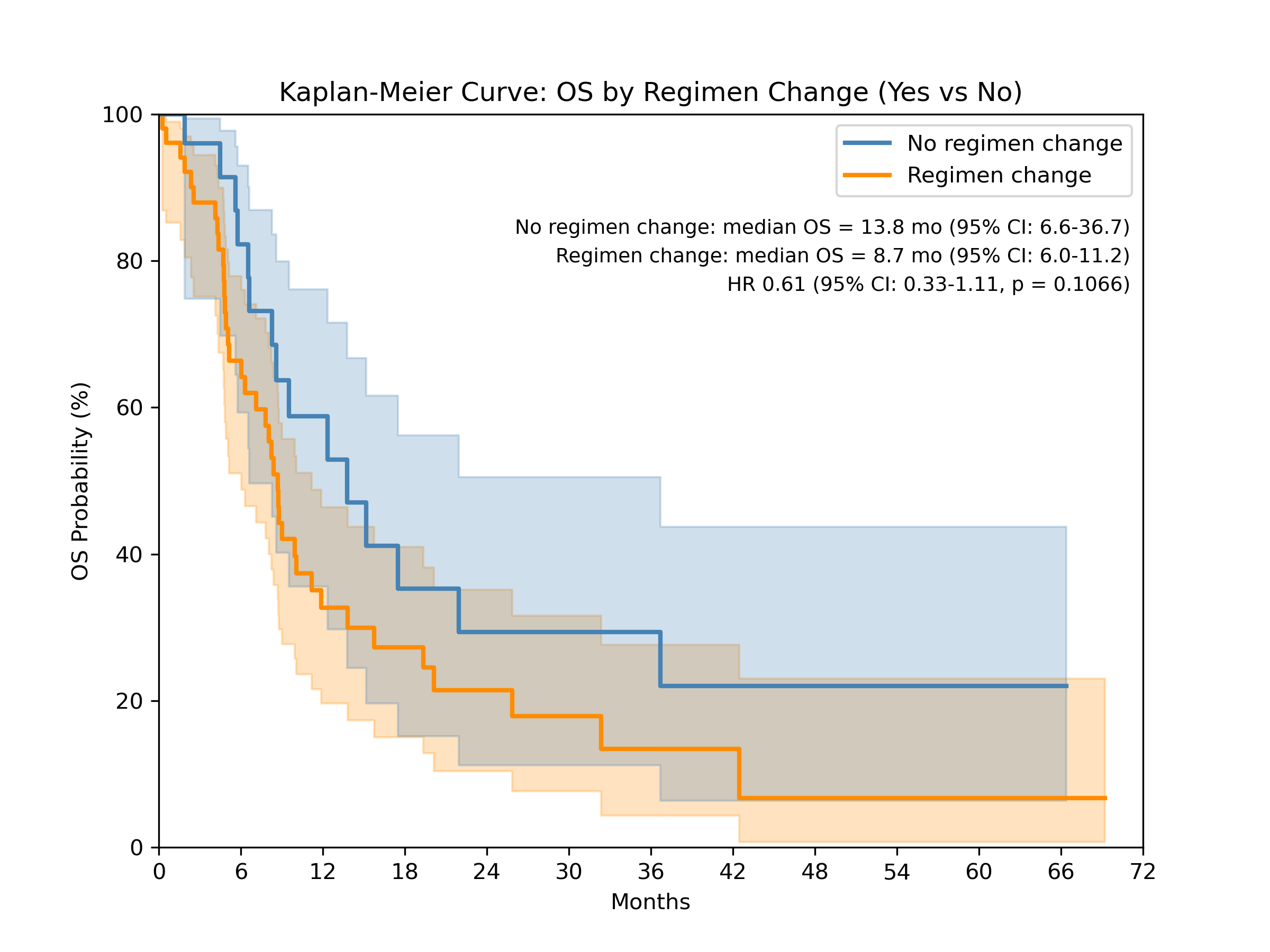

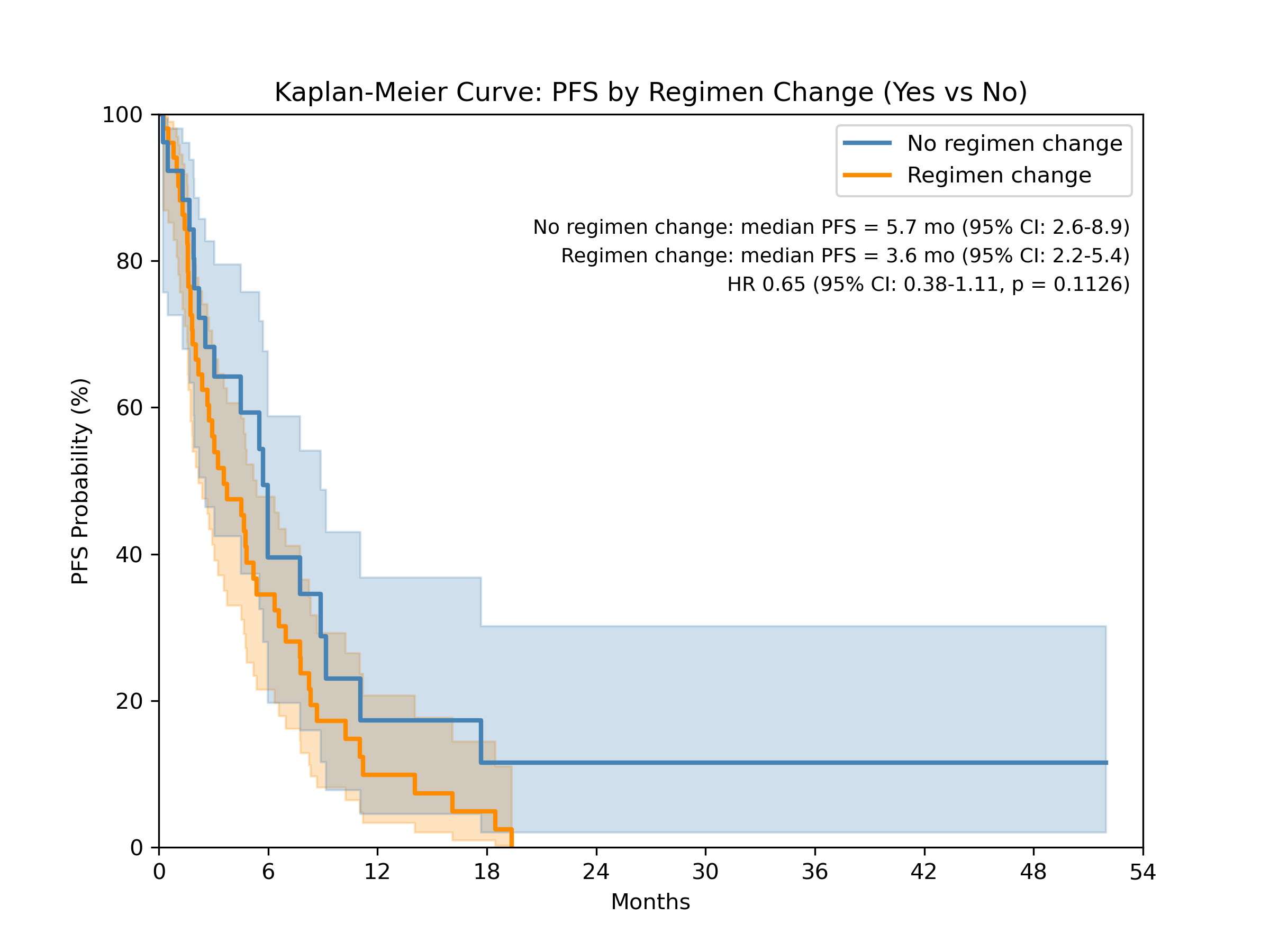


B

C

D


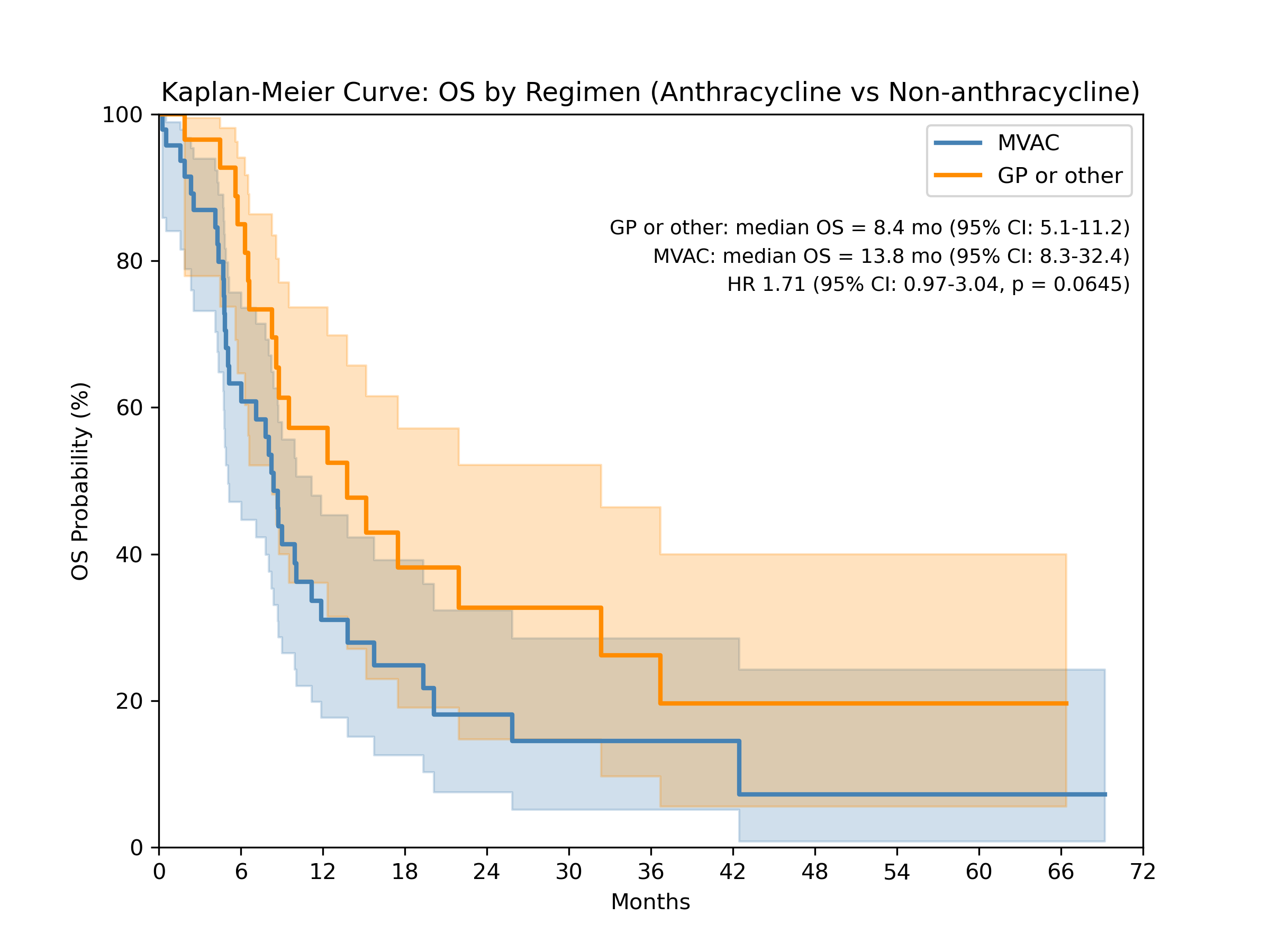

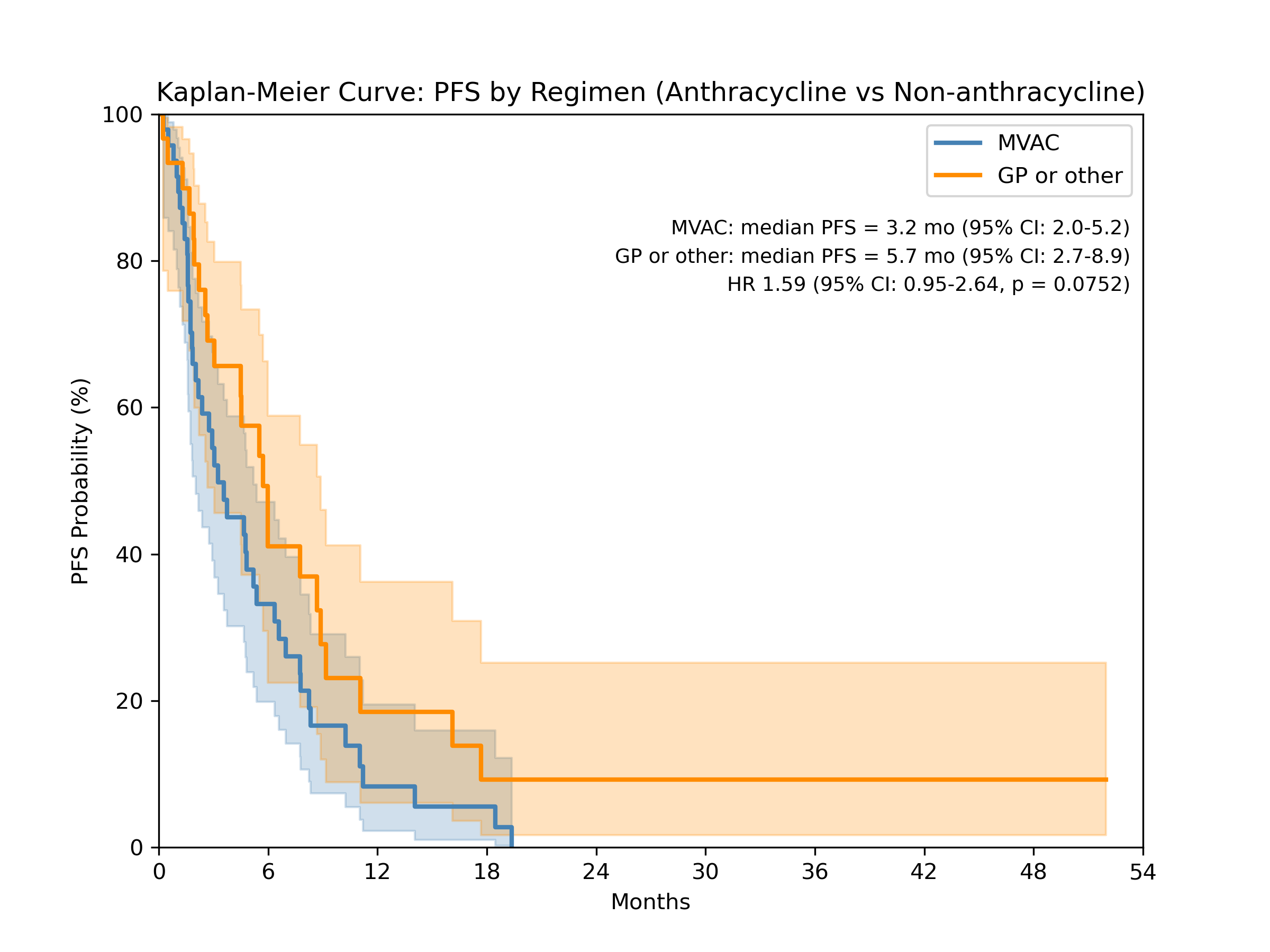


A

**S2 Fig. Kaplan–Meier curves for OS and PFS after platinum rechallenge according to the timing of immunotherapy exposure.**

A

B
